# Rapid recovery of homozygous *Pr* gene introgression lines in Indian tropical cauliflower backgrounds through combined use of morphological and molecular markers

**DOI:** 10.3389/fpls.2025.1609917

**Published:** 2025-09-29

**Authors:** Shrawan Singh, Sandeep Kumar, Vinay Verma

**Affiliations:** ^1^ Division of Vegetable Science, Indian Council of Agricultural Research (ICAR) Institute, New Delhi, India; ^2^ Indian Council of Agricultural Research (ICAR) Institute Regional Station, Kullu, Himachal Pradesh, India

**Keywords:** anthocyanin, *Brassica oleracea* var. *botrytis* L., developmental transitions, gene introgression, marker assisted selection

## Abstract

Anthocyanin is a groups of secondary metabolites which are strong antioxidants. Biofortificaiton of commonly used foods for anthocyanin enhance the chances of its intake and enjoy health benefits by common people. The study aimed at rapid anthocyanin biofortification of tropical cauliflower by combining morphological and marker-assisted selections. Two tropical varieties, Pusa Ashwini (PA) and Pusa Kartiki (PK), were crossed with the donor KTPCF-1 (or PPCF-1) of the snowball group. The F_2_, BC_1_F_1_, and F_2:3_ populations from PA/PPCF-1 and PK/PPCF-1 supported a single dominant gene (*Pr*) for purple colour in both the seedling apical and curd portions. The F_2_ and BC_1_F_2_ plants were first selected for purple curd and morphological traits specific to tropical cauliflower, namely, semi-erect leaf habit, October–November maturity, and tropical flowering habit. A total of 40 and 30 purple curding plants were selected in F_2_ and BC_1_F_2_, respectively. Visual detection of homozygous and heterozygous purple plants was unreliable. Thus, these 70 plants were screened using two codominant (BoMYB2m and BoMYB4m) and one dominant (BoMYB3m) markers for forward selection. A total of 19 plants in F_2_ and 21 plants in BC_1_F_2_ were found homozygous for the *Pr* gene, of which 13 F_2_ plants and all 21 BC_1_F_2_ plants produced sufficient seeds to advance F_2:3_ and BC_1_F_2:3_, respectively. The progenies showed a significant increase in total anthocyanin content. The marker-assisted selection (MAS)-derived *PrPr* progenies, namely, PC2304-21, PC2304-93, PC2304-64, PC6704-16, and PC6704-36, were the most promising with higher curd yield (>17.2 t/ha), hence advanced to F_3:4_. These tropical-type progenies are of immediate breeding use for anthocyanin-rich varieties/hybrids to harness the associated benefits in the tropics.

## Introduction

Cauliflower (*Brassica oleracea* var. *botrytis* L.) is one of the most important cole vegetables being grown in 97 countries on 1.37 million ha with an annual production of 26.06 million metric tonnes (FAOSTAT, 2023). China and India are the two largest producers, with global shares of 37.06% and 36.71%, respectively. It has spread both temporally and spatially due to the evolution of tropical types (Singh et al., 2022), which have tolerance to hot and humid climates (Swarup and Chatterjee, 1972). This tropical cauliflower can set seeds freely at moderate temperatures and develop curd at temperatures between 16°C and 30°C (Singh and Kalia, 2021), thus expanding the horizons of cauliflower cultivation.

The consumers’ traditional preference is inclined towards white (or bleached) cauliflower, which contains only glucosinolates as the potential anticancer compound. However, purple is due to the accumulation of anthocyanin in the curd portion. This is a novel, eye-catching, brilliant-coloured cauliflower and is becoming popular for its health benefits (Mattioli et al., 2020; Singh et al., 2020). Anthocyanins are known potential agents against atherosclerosis, cardiovascular disease, cancer proliferation, and neurodegenerative diseases (Youdim et al., 2000; 2002; Lazze et al., 2003; Rossi et al., 2003). They play a role in guarding against age-related declines in cognitive performance (Cho et al., 2003), diminishing pulmonary inflammation (Jennings et al., 2014), and managing type 2 diabetes (Li et al., 2015). Clinical reviews showed that anthocyanin supplementation significantly improved low-density lipoproteins and reduced cholesterol levels among diseased individuals (Wallace et al., 2016) and significantly improved glycaemic parameters and lipid profile in type 2 diabetes patients (Mao et al., 2023; Koziowska and Nitsch-Osuch, 2024). Dietary supplementation significantly reduced pro-inflammatory markers in type 2 diabetes individuals (Nikbakht et al., 2021) and reduced platelet aggregation and modulated mechanisms involved in thrombogenesis in the obese/overweight population (Thompson et al., 2017). However, anthocyanins remain for only up to 6 hours in the body (Wallace et al., 2016) and do not store for a longer period; hence, regular intake is necessary. China is the only country that has published a dietary reference intake of anthocyanins (50 mg/day) in its national nutrition guidelines. Except for this, there is no prescribed limit for anthocyanins, but they are active as low as 7.5 mg/day, and there is no reported harmful effect noticed up to 300 mg/day intake in Sprague–Dawley rats (Suresh and Vellapandian, 2024) and up to 320 mg/day in human subjects with hypercholesterolaemia (Mattioli et al., 2020). Thus, anthocyanin biofortification of large-volume and year-round-available vegetables such as cauliflower is a significant, promising option in ensuring a consistent supply and addressing various human health challenges (Lila, 2004). Furthermore, purple cauliflower has cyanidin 3(-coumaryl-caffeyl)glucoside-5-(malonyl)glucoside as the prominent anthocyanin, which is reported as a potent anti-diabetic agent (Wedick et al., 2012).

The growing of colourful cauliflowers is confined to the winter season in sub-tropical regions due to the low temperature (10°C–16°C) requirement for curd formation. Kalia et al. (2018) successfully introgressed the *Or* gene from snowball group genotype ‘1227’ of orange cauliflower into Indian cauliflower, which confers increased β-carotene level, and thus orange or orange-yellow colour to cauliflower curd. In similar lines, attempts were required to introgress the *Pr* gene from the snowball group into the background of tropical cauliflower. This will allow the temporal and spatial expansion of the anthocyanin-rich cauliflower production for consumers’ well-being.

Cauliflower is thermosensitive for developmental transitions, which are crucial for economic crop production as well as seed production (Luhana et al., 2023). The novel mutants, such as the *Or* gene, delay the onset of curding and flowering transitions in cauliflower (Crisp et al., 1975; Chandel et al., 2025). Since the impact of the *Pr* gene has not been investigated before, it is necessary to study its effects during introgression in Indian tropical cauliflower.

Chiu et al. (2010) reported the *Pr* gene as a semi-dominant gene and reported two codominant markers (namely, BoMYB2m and BoMYB4m) and one dominant marker (BoMYB3m) to distinguish homozygous and heterozygous plants. The codominant markers discriminate between homozygous (*PrPr*) and heterozygous (*Prpr*) plants; hence, they are useful in foreground selection in marker-assisted selection (MAS). MAS is the most effective strategy to identify the desirable homozygous genotype through foreground selection in segregating materials (Collard and Mackill, 2008). The present study aimed at foreground selection for the *Pr* gene since the donor genotype is an agronomically superior variety but from a different maturity group (i.e., snowball group) (I.A.R.I., 2018). The use of markers has increased substantially due to the development of high-throughput markers and gene-based markers, which can be used in a cost-effective manner.

Thus, the present study aimed at introgression of the *Pr* gene in Indian cauliflower using MAS and characterised the promising *Pr*-introgressed F_2:3_ progenies for anthocyanin content and morphological characteristics.

## Materials and methods

### Development of breeding materials

**Figure 1:**
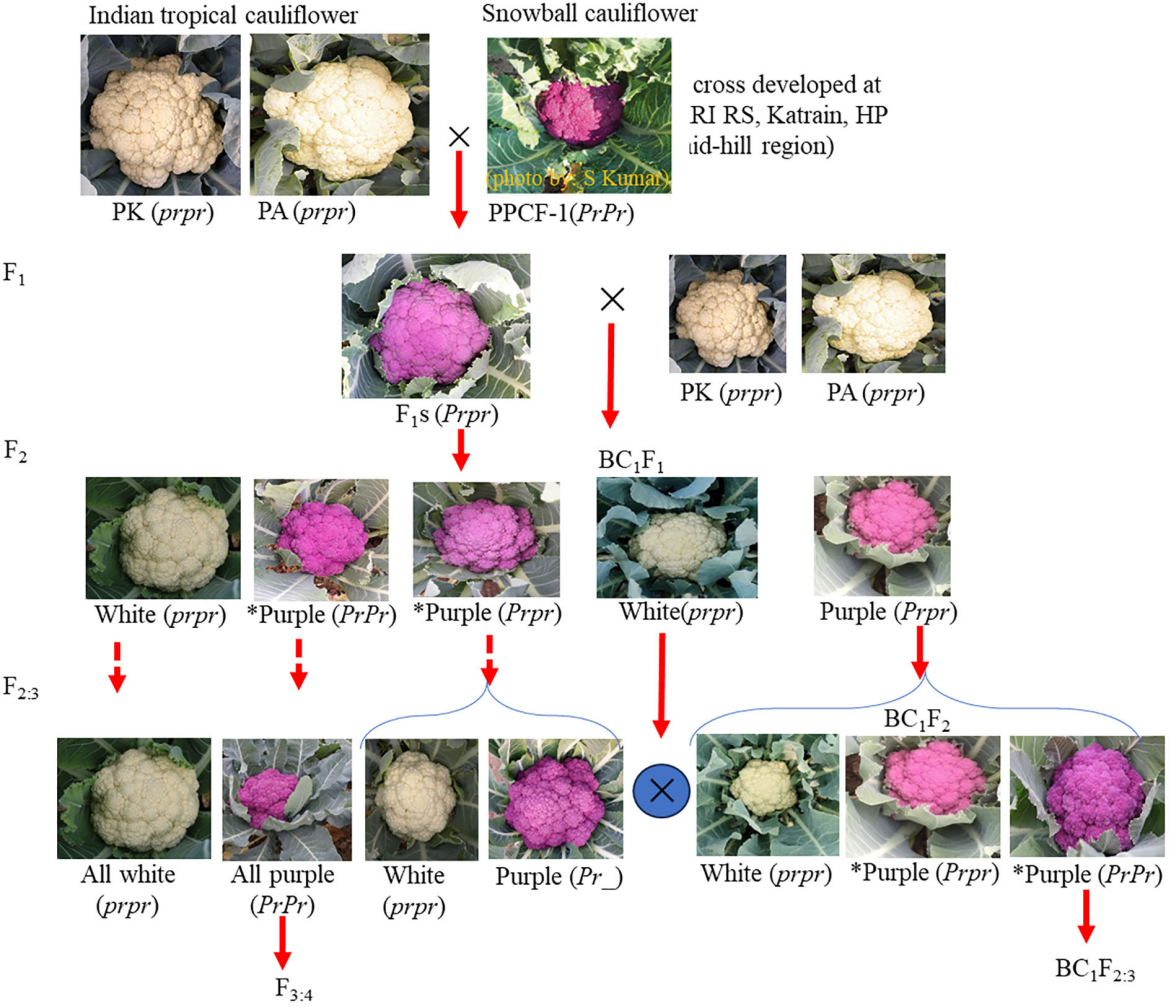
Segregation for curd colour observed during introgression of *Pr* gene in Indian tropical cauliflower background.

The development of breeding materials is shown in **Figure 1**. Two white curd varieties, namely, Pusa Ashwini (PA) and Pusa Kartiki (PK) (as female parent) of the early maturity group of Indian tropical cauliflower, were crossed with purple cauliflower line KTPCF-1 (PPCF-1) (as male parent) of the snowball group. The snowball group forms curd at 10°C–16°C, while the early group forms at 20°C–27°C. Unlike tropical varieties ‘PA’ and ‘PK’, ‘PPCF-1’ does not flower satisfactorily in plains; hence, F_1_s were generated at IARI Regional Station, Katrain, Kullu, HP, in 2019–2020. The synchronisation in flowering between ‘PPCF-1’ and recipient varieties ‘PA’ and ‘PK’ was obtained by adjusting the sowing time.

The F_1_ seeds (‘PA’ × ‘PPCF-1’; ‘PK’ × ‘PPCF-1’) were transported to IARI, New Delhi, and sown in the nursery in August. Two F_1_ plants from each set were selfed by bud pollination to generate F_2_s, and also F_1_ was backcrossed (as female parent) with ‘PA’ and ‘PK’ (as male parent) to obtain BC_1_F_1_. The F_2_ and selected purple BC_1_F_1_ plants were advanced to F_2:3_ and BC_1_F_2_, respectively.

### Morphological selection in F_2_ and BC_1_F_2_

The F_2_ and BC_1_F_2_ plants were first selected for i) purple curd colour, followed by morphological traits, i.e., ii) semi-erect leaf, iii) October–November curd maturity, and iv) tropical flowering habit (flowering in December–January) (Singh and Kalia, 2020). White curding plants were also advanced for evaluation purposes. A total of 40 F_2_ plants were selected in both sets (i.e., PA/PPCF-1 and PK/PPCF-1). Similarly, 30 plants in BC_1_F_2_ were also selected for marker analysis.

### Marker-assisted selection

**Figure 2:**
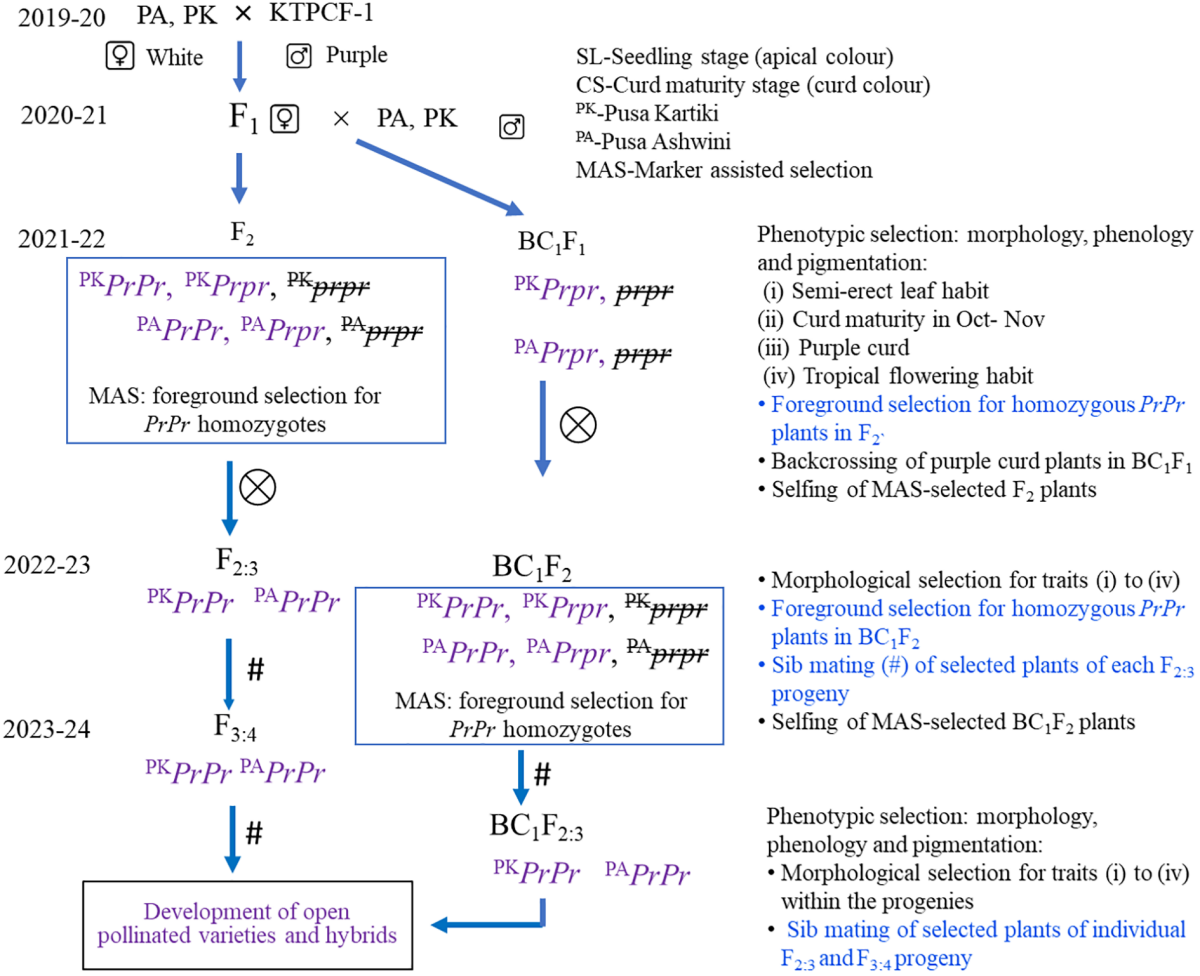
Procedure for marker-assisted selection of *Pr* gene introgression progenies in Indian tropical cauliflower background. MAS, marker-assisted selection.

The procedure for marker-assisted selection is presented in **Figure 2**. The F_2_ plants were first observed for purple curd colour, followed by selection on the basis of criteria associated with the plant type of tropical cauliflower, i.e., semi-erect leaf, October–November curd maturity, and tropical flowering habit. A total of 48 F_2_ plants were selected, including 40 purple curding and eight white curding plants for MAS. The F_2:3_ progenies from these selected homozygous (*PrPr*) F_2_ plants were advanced to F_3:4_. Similarly, the 18 morphologically selected BC_1_F_2_ plants from PA and 12 plants from PK were also taken for foreground selection with *Pr* gene-linked markers. The identified homozygous (*PrPr*) BC_1_F_2_ plants were advanced to BC_1_F_2:3_. The progenies from these 70 plants were tested in F_2:3_ and BC_1_F_2:3_ generations for seedling apical colour and curd colour to confirm their zygosity level.

The genomic DNA from the leaf samples was isolated using the cetyltrimethylammonium bromide (CTAB) method (Murray and Thompson, 1980). The extracted DNA was treated with RNase as described by Rakshita et al. (2021). The DNA quality and quantity were checked using a 0.8% agarose gel and Nanodrop (Shimadzu, Kyoto, Japan). Three polymerase chain reaction (PCR)-based *Pr* gene-linked primers, namely, BoMYB2m, BoMYB3m, and BoMYB4m (from Chiu et al., 2010), were synthesised from Integrated DNA Technologies (IDT) through JP Scientific, India (**Supplementary Table S1**). These primers were first tested in three parental lines and seven random purple curd F_2_ plants. Furthermore, the primers were screened using the genomic DNA of all 48 F_2_ plants by following the PCR programme as described by Singh et al. (2022). The amplicons were resolved through gel electrophoresis using a 3% agarose gel and visualised in a gel documentation system (Bio-Rad, Hercules, California, USA).

### Field evaluation of breeding materials

The populations (parental lines, F_1_, F_2_, and BC_1_F_1_) were evaluated in the Research Farm, IARI, New Delhi, during 2021–2022. The crop was raised following standard crop-growing practices (Singh and Sharma, 2001). The location of the research farm is 28.63 N, 77.15 E, and is 228.61 m above mean sea level. The monthly mean temperature during the crop period ranged from 12.2°C to 25.0°C. Similarly, all the F_2:3_ progenies and BC_1_F_2_ were raised for evaluation in the subsequent year (i.e., 2022–2023) by following the standard crop-growing practices (Singh and Sharma, 2001).

### Characterisation for morphological traits and developmental transitions

A total of 13 MAS-derived homozygous F_2:3(purple)_ progenies, including six from PA × PPCF-1 and seven from PK × PPCF-1, were raised in paired rows (15 plants/row) in a randomised block design with three replications. The observations on seven morphological traits, i.e., leaf length, leaf width, plant height, plant spread, gross plant weight, marketable curd weight, and marketable curd yield, were recorded from 10 random plants. Furthermore, days taken for curd initiation, marketable curd maturity, bolting, flowering, and seed harvesting stages were recorded from the remaining 10 plants to assess their closeness for developmental transitions to the tropical varieties PA and PK. Parental lines (PA, PK, and PPCF-1) and ‘Graffiti’ (a purple cauliflower variety, Syngenta) were used as the references. The mean value was used for comparison.

### Total anthocyanin analysis

Curd samples measuring approximately 50 g were harvested per selected plant and homogenised, from which 500 mg per plant was analysed for total anthocyanin content using the differential pH method as described by Scalzo et al. (2008).

### Statistical analysis

The χ^2^ method was used to test the goodness of fit of the observed ratios for seedling apical colour and curd colour to the theoretically expected ratio in the segregating populations (F_2_, BC_1_, and F_2:3_) (Panse and Sukhatme, 1967). The data from morphological and developmental transitions were analysed for the level of significance using the ‘agricolae’ package of RStudio. The normal distribution curve for anthocyanin content in plant materials was analysed using population size (*N* = 90) and the Microsoft Excel^®^ software (Singh et al., 2020).

## Results

### Phenotypic segregation for apical colour and curd colour traits

The F_1_ plants produced purple colour seedling apical leaves and marketable curd. The intensity of colour was less than that of the parental line ‘PPCF-1’, and it was absent in both ‘PA’ and ‘PK’ (**Table 1**).

**Table 1:** Segregation of F_2_, BC_1_, and F_2:3_ from PA/PPCF-1 and PK/PPCF-1 crosses for seedling apical colour and marketable curd colour in Indian cauliflower backgrounds.

Cross	Progeny group	Apical colour at seedling stage				p-Value	Curd colour				p-Value
		Total plants/progenies	Purple	White	χ^2^ value (cal)		Total plants	Purple	White	χ^2^ value (cal)	
PA × PPCF-1	F_2_	531	385	153	3.39	0.065	365	267^$^	98^$^	0.67	0.41
	BC_1_ F_1_ (F_1_ × PA)	278	128	150	1.74	0.19	127	59	68	0.64	0.42
	F_2:3 (purple)_	1,513 (213)	1,140	373	0.09	0.76	1,296 (148)	1,002	294	3.7	0.054
	F_2:3 (purple, non-segregating)_	1,576 (48)	1,576	0			1,492 (48)	1,492	0		
	F_2:3 (white)_	836 (34)	0	836			183(16)	0	183		
	BC_1_F_2_ (of F_1_ × PA)	331	239	92	1.37	0.24	279	198^$^	81	2.41	0.12
PK × PPCF-1	F_2_	438	313	125	2.93	0.087	278	167^$^	71^$^	2.96	0.085
	BC_1_ F_1_(F_1_ × PK)	276	122	154	3.71	0.054	143	76	67	0.57	0.45
	F_2:3 (purple)_	1,572 (97)	1,205	367	2.29	0.13	1,090 (86)	844	246	3.44	0.064
	F_2:3 (purple, non-segregating)_	1,360 (61)	1,360	0			1,118 (61)	1,118	0		
	F_2:3 (white)_	556 (43)	0	556			13	0	171		
	BC_1_F_2_ (of F_1_ × PK)	378	268	110	3.39	0.066	322	227^$^	95	3.48	0.062

Values in parentheses are the number of progenies. 1) χ^2^ values were calculated based on goodness-of-fit ratios of 3:1 except for BC_1_F_1_ generations, where they are 1:1. 2) The separation of F_2:3_ progenies into purple [F_2:3(purple)_] and [F_2:3(purple non-segregating)_] is based on the observation for curd colour from F_2:3_, not on F_2_ population. 3) The separation of F_2:3(white)_ was based on observations for curd colour in F_2_ population.

NS, non-segregating; MAS, marker-assisted selection.

^$^Selected plants taken for MAS in F_2_ and BC_1_F_2_.

*Genetics of purple apical colour:* The F_2_ segregation for both crosses was not significantly different from the expected 3:1 ratio (**Table 1**). The F_2:3_ families were split into those that continued to segregate, which similarly followed the expected 3:1 ratio and those in which apical leaves were uniformly purple (**Table 1**). No segregation was observed in the F_2:3(green)_ progenies. Both backcross-derived crosses followed the expected 1:1 ratio at BC_1_F_1_ and the 3:1 ratio at BC_1_F_2_ (**Table 1**).

*Genetics of purple curd colour*: Segregation for curd colour followed similar patterns. Both F_2_ populations followed the expected 3 (purple):1 (white) segregation ratios (**Table 1**). A number of F_2:3_ families were fixed for either purple or white curd and showed no segregation, but the progenies within segregating families also followed the 3:1 ratio (**Table 1**). For backcross-derived material, both BC_1_F_1_ crosses followed the expected 1:1 ratio, while the BC_1_F_2_ progenies segregated as 3:1. All seedlings with apical colour developed into plants with purple curd, showing that the *Pr* gene controls purple pigmentation throughout plant development.

Plants with bicolour curd phenotype (having two colours in a single curd) were observed in both sets of F_2_ populations (**Supplementary Figure S1**). PK × PPCF-1 had a greater number of such plants (11) than PA × PPCF-1/PA (9). PK/PPCF-1 had eight plants with purple + white curd and three plants with intense purple + light purple. There were five plants with purple + white and four plants with intense purple + light purple for PA/PPCF-1. The bicolour curd plants also had purple apical colour pigmentation.

### Marker-assisted selection using foreground marker

**Figure 3:**
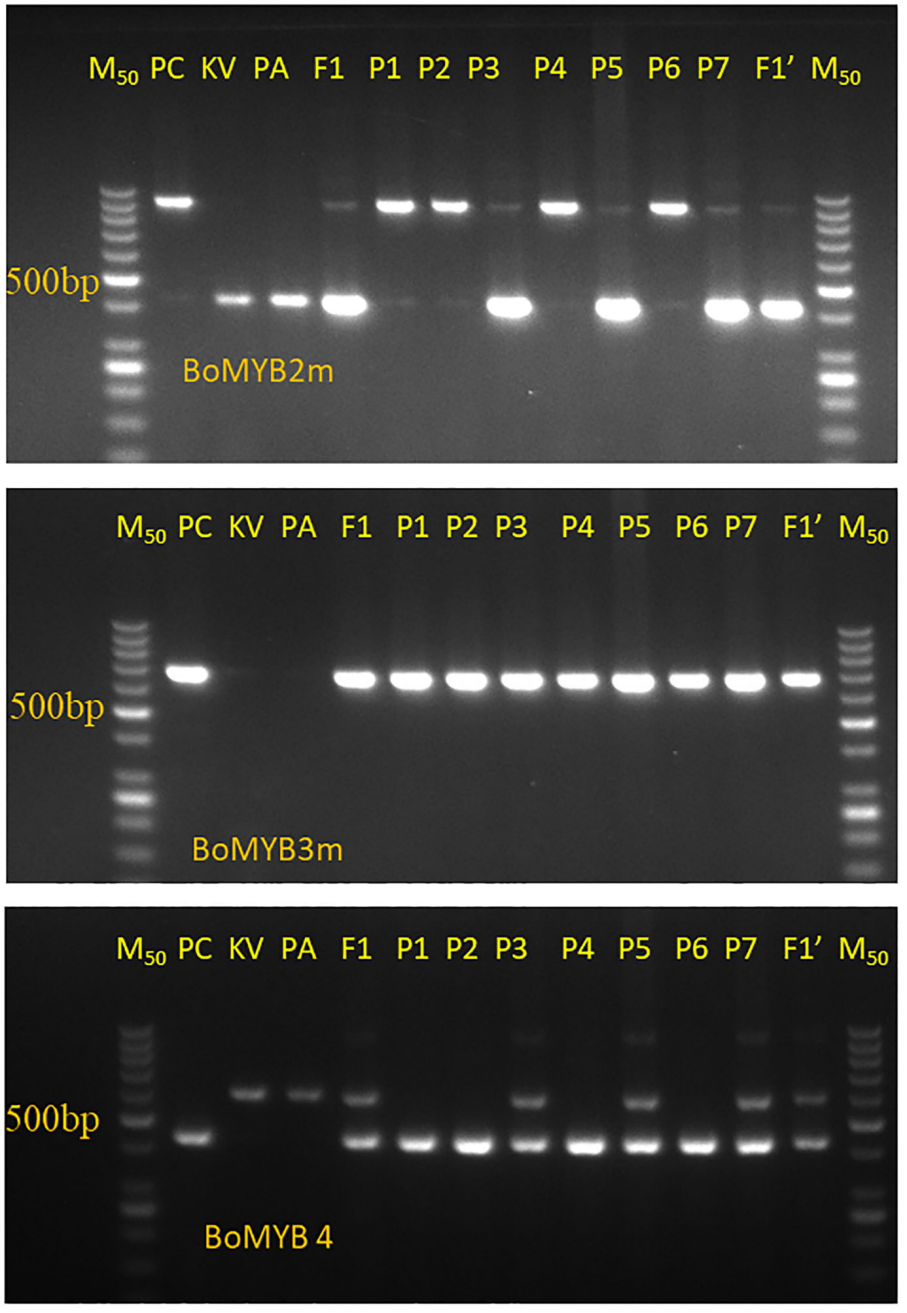
Amplification pattern of three *Pr* gene-linked markers, namely, BoMYB2m, BoMYB3m, and BoMYB4m, in parental lines (PC or PPCF-1, PK, and PA), their F_1_s (F_1_-PA/PPCF-1 and F_1_′-PK/PPCF-1), and seven selected purple F_2_ plants (Sl. Nos. P1 to P7).

**Figure 4:**
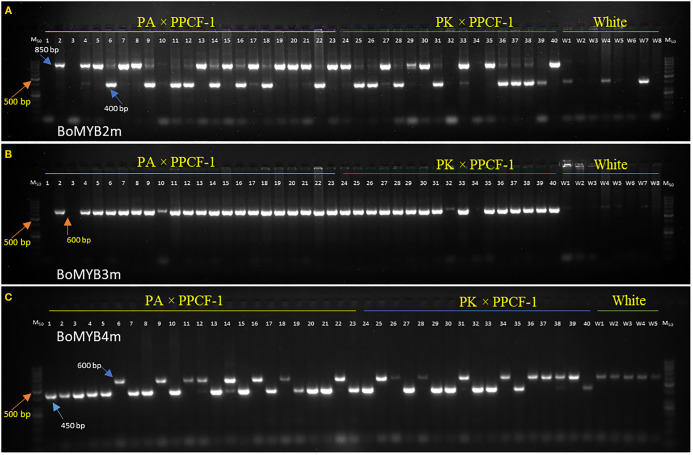
Screening of *Pr* gene-specific primers BoMYB2m **(A)**, BoMYB3m **(B)**, and BoMYB4m **(C)** in F_2_ populations from PA × PPCF-1 (Sl. No. 1–23 plants), PK × PPCF-1 (Sl. No. 24–40 plants), and white cauliflower genotypes (Sl. Nos. W1–W8, up to W1–W5 in case of BoMYB4). M_50_ is a marker of 50 base pairs.

The results of PCR amplification of three *Pr* gene-linked markers (BoMYB2m, BoMYB3m, and BoMYB4m) in three parental lines, two F_1_s, and seven random purple curding F_2_ plants are presented in **Figure 3**. All three markers were polymorphic for the *Pr* locus. BoMYB2m and BoMYB4m were codominant, while BoMYB3m was dominant (**Figures 4A–C**). The screening of 40 selected early maturing purple curding F_2_ plants with BoMYB2m (**Figure 4A**) and BoMYB4m (**Figure 4C**) revealed 19 as common homozygous and 16 as heterozygous for the *Pr* gene. BoMYB4m revealed 23 F_2_ plants as homozygous. Five plants (Nos. 1, 3, 10, 32, and 34) did not amplify a proper amplicon with BoMYB2m. BoMYB2m amplified two amplicons of 850 bp (in purple) and 400 bp (in white), while BoMYB4m produced 450-bp (in purple) and 600-bp (in white) amplicons. BoMYB2m amplified a prominent band of 850 bp in purple homozygous plants. Heterozygous purple plants amplified a prominent band of 400 bp along with a faint band of 850 bp. BoMYB2m amplified an amplicon of 400 bp in eight white plants, and BoMYB4m amplified an amplicon of 600 bp in five white curding plants. BoMYB3m amplified an amplicon of 650 bp only in all the purple plants (**Figure 4B**). Out of 19 MAS-derived homozygous F_2_ plants, only 13 plants could produce sufficient seeds (F_2:3_). Furthermore, 30 BC_1_F_2_ plants having purple curds and matched with the recurrent parents (PA and PK) for the key morphological traits (i.e., semi-erect leaf, October–November curd maturity, and tropical flowering habit) were screened using BoMYB4m markers and identified 11 plants from PA/PPCF-1 cross and 10 plants from PK/PPCF-1 as homozygous for the *Pr* locus (**Supplementary Figure S2**). Furthermore, the observations from the F_2:3_ and BC_1_F_2:3_ progenies of these 70 plants for seedling apical colour and curd colour confirmed their zygosity level and validated the marker results (**Supplementary Table S2**).

### Morphological traits and developmental transitions of selected F_2:3_ progenies

The 13 MAS-selected homozygous F_2:3_ progenies showed a significant variation in morphological and yield traits (**Supplementary Table S2**). All the plants from these 13 progenies had purple curds, confirming their homozygous state. Leaf length ranged from 36.2 to 48.0 cm and leaf width from 10.1 to 18.3 cm. Plant height (55.7 cm) and plant spread (53.3 cm) were maximum in PC2304-21. This progeny also had maximum gross plant weight (1,574.7 g) and marketable curd weight (817.7 g). The marketable curd yield level ranged from 15.8 to 25.7 t/ha. Two F_2:3_ progenies from each PK/PPCF-1 and PA/PPCF-1 had yield levels higher than those from PK and PA, respectively.

The selected F_2:3(purple)_ progenies from both sets, PA × PPCF-1 and PK × PPCF-1, showed significant earliness than ‘Graffiti’ and ‘PPCF-1’. The progenies were closer to the recipient varieties PA and PK (**Table 1**). It was similar for the case of both F_1_s. ‘Graffiti’ had the maximum days for each of the observed plant stages in the Delhi condition, followed by the immediate donor parent ‘PPCF-1’. The progenies from PA/PPCF-1—PC6704-16 (109.3 ± 2.5 DAS), PC2304-66 (113.3 ± 2.5 DAS), and PC2303-21 (117.0 ± 2.0 DAS)—were the earliest for curd maturity.

### Anthocyanin content

**Figure 5:**
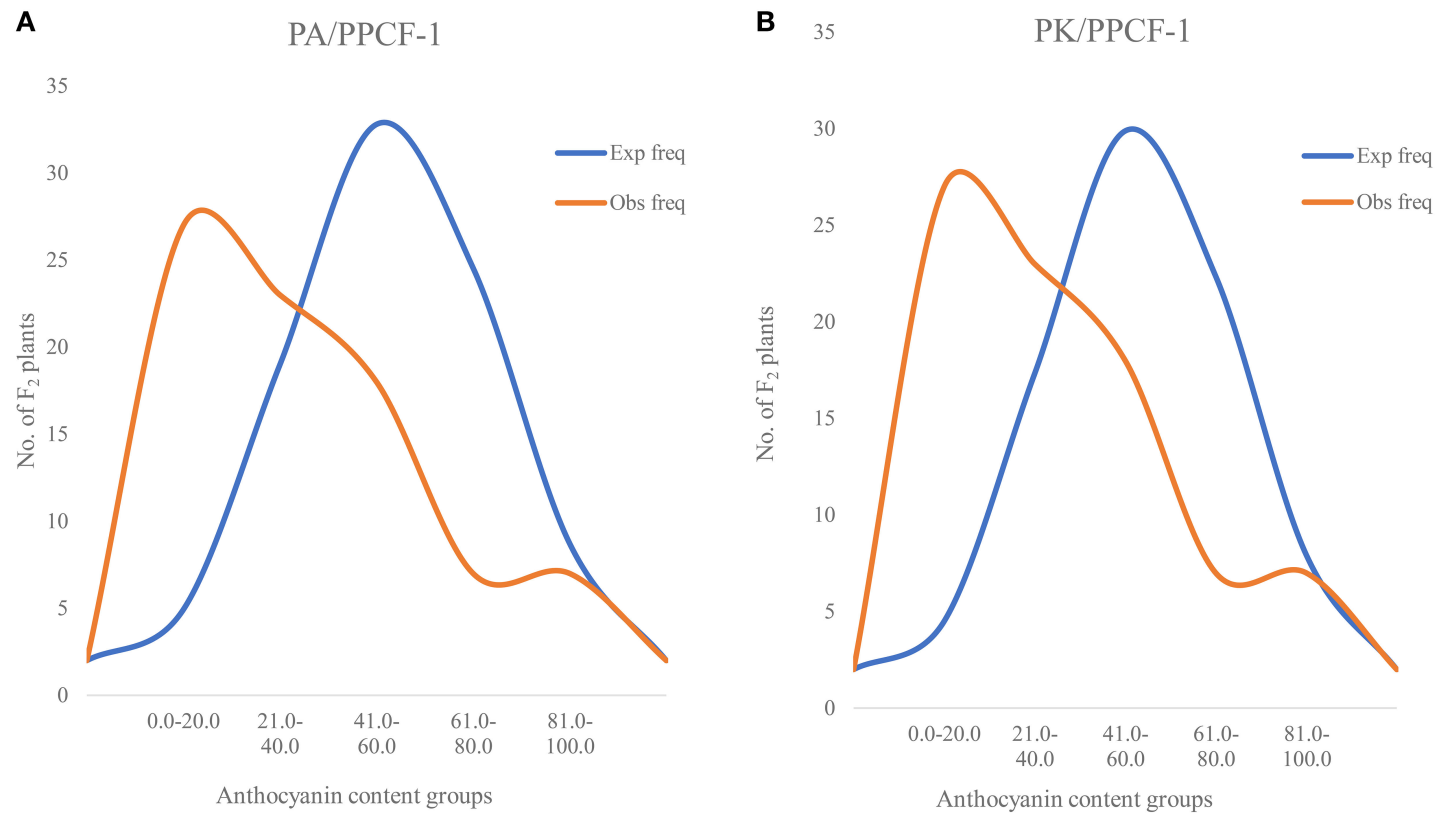
Observed distribution of anthocyanin content (mg/100 g fw) in selected F_2_ plants from PA/PPCF-1 (*N* = 90) **(A)** and PK/PPCF-1 (*N* = 82) **(B)**.

*Anthocyanin in F_2_ population:* In F_2_ populations, anthocyanin analysis in 90 F_2_ plants (only with purple curd) from PA × PPCF-1 resulted in a range of 6.5 to 94.6 mg/100 g fw, while it varied from 2.34 to 97.5 mg/100 g fw in F_2_ plants from PK × PPCF-1 (**Figures 5A, B**). A total of 22 and 33 F_2_ plants from both sets had anthocyanin content higher than 43.0 mg/100 g fw. In white varieties, it was negligible, i.e., from 0.2 to 0.83 mg/100 g fw, and in both parental lines PA (0.3 mg/100 g fw) and PK (0.2 mg/100 g fw).

**Figure 6:**
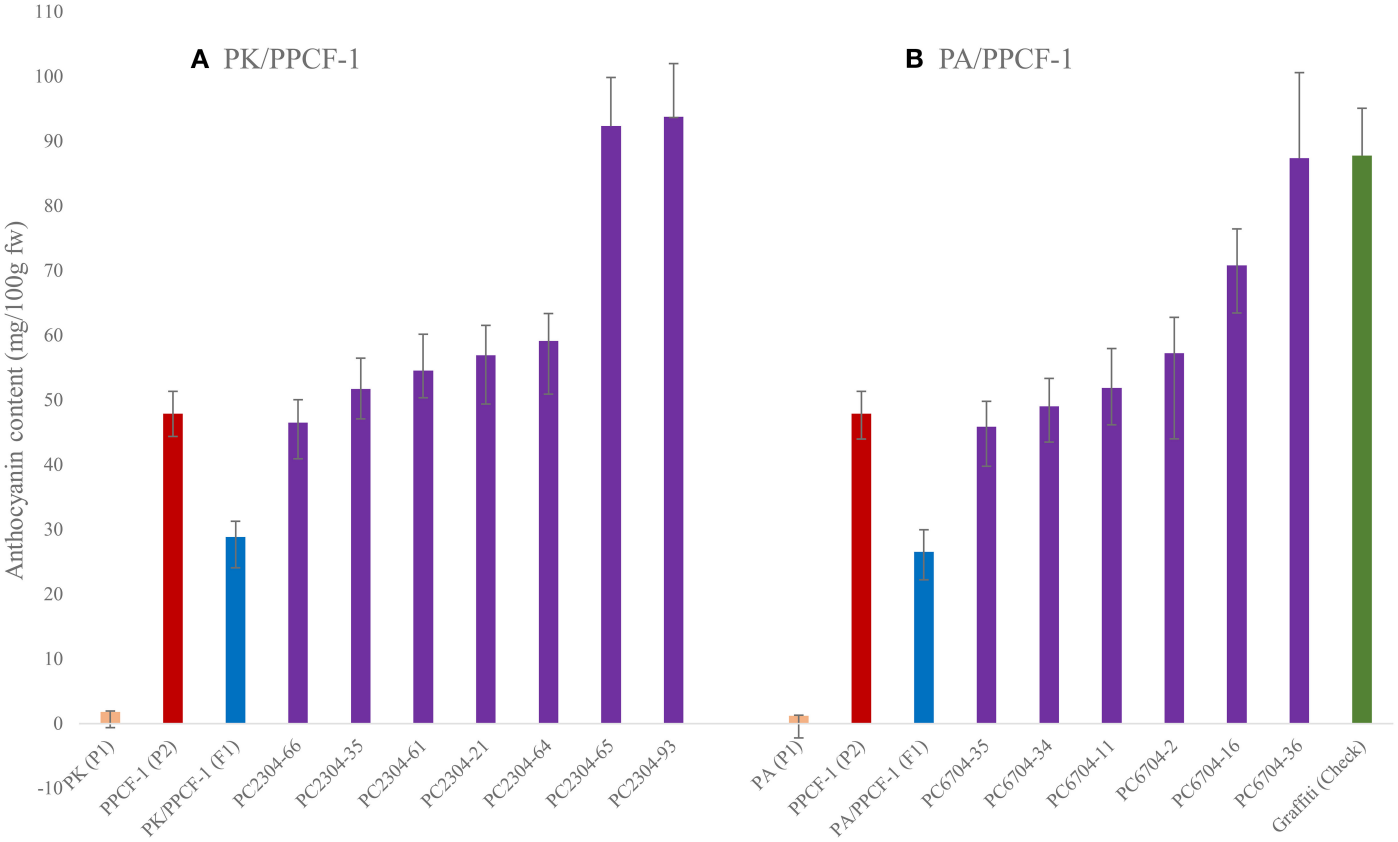
Anthocyanin content (with standard error) in selected promising F_2:3_ progenies of PK/PPCF-1 **(A)** and PA/PPCF-1 **(B)**. Orange bar, parents; red bar, donor parent; blue bar, F_1_s; purple bar, F_2:3_ progenies; green bar, Graffiti (Check).

*Anthocyanin in F_2:3_ progenies:* Anthocyanin content in 13 selected F_2:3_ progenies is shown in **Figure 6**. The most promising progenies for anthocyanin in the curd portion were PC2304-93 (93.78 mg/100 g fw) and PC2304-65 (92.34 mg/100 g fw) from PK/PPCF-1, and PC6704-36 (87.37 mg/100 g fw) and PC6704-16 (70.8 mg/100 g fw) from PA/PPCF-1. The selected progenies had 57.5 to 115.8 times higher anthocyanins in the PK/PPCF-1 set and 37.9 to 72.2 times higher anthocyanins in the PA/PPCF-1 set than white curding recipient varieties PA and PK, respectively. Furthermore, 11 progenies had higher anthocyanin than the donor parent PPCF-1 (53.65 mg/100 g fw).

## Discussion

Breeding biofortified varieties without disrupting the general phenology and adaptation is the key to success. In cauliflower, introgression of the *Or* gene in the homozygous state (*OrOr*) resulted in morphological and yield penalties, which restricted its commercial potential in the heterozygous state only (Crisp et al., 1975; Kalia et al., 2023). The primary aim of this study was to transfer the *Pr* gene into a tropical cauliflower background within a short period. The study has been successful in the development of progenies, which were true-breeding for purple curd (*PrPr*), with equivalent yield and development to typical white cauliflower. The use of the *Pr* gene-linked molecular markers could reduce the time required for developing the desirable homozygous *PrPr* progenies to 3 to 4 years.

The biofortification of cauliflower for anthocyanins is an important step towards fighting against non-communicable diseases (NCDs), which are the biggest challenge to global health, as they account for almost 62% of all deaths in the Southeast Asian region (who.int). Globally, low intake of fruits and vegetables (F&V) is a risk factor for NCDs, which account for an estimated 1.7 million deaths, including gastrointestinal cancer (14%), ischaemic heart disease (11%), and stroke (9%). F&Vs are the most promising dietary agents to combat these widespread NCDs. Anthocyanins act as antioxidants and play a wide spectrum of health-related activities against NCDs, such as cancer, cardiovascular diseases, type 2 diabetes, and obesity (Meiers et al., 2001; Kozłowska and Nitsch-Osuch, 2024).

The commercial white cauliflower contains sinigrin and glucoerucin glucosinolates as the major health compounds (Vanlalneihi et al., 2020; Singh et al., 2025). However, other compounds such as anthocyanins are also present in cauliflower germplasm (Kalia et al., 2023).

Thus, the biofortification of cauliflower with anthocyanin through introgression of the *Pr* gene is an important step to contribute to public health. The purple colour of cauliflower occurred due to a spontaneous mutation in a single gene in European-type cauliflower (Chiu et al., 2010). Thus, the *Pr* gene enhanced the functional capacity of the crops twice due to the glucosinolate + anthocyanin combination. The glucosinolates are also reported in the *Pr* gene donor purple cauliflower ‘Graffiti’—total glucosinolates (8.260 ± 0.16 µmol/g dw), sinigrin (4.374 ± 0.07 µmol/g dw), and glucoiberin (1.915 ± 0.0.02 µmol/g dw)—which were reduced during conventional cooking by 69.2%, 71.2%, and 52.5%, respectively (Kapusta-Duch et al., 2016). However, the glucosinolates that are similar to anthocyanins are mainly affected by genotype and less by environment and agronomic practices, thereby warranting further investigation in the *Pr* gene introgression lines of cauliflower.

The commercial varieties (i.e., Graffiti from Syngenta, PPCF-1 from IRARI RS, Katrain, HP, India) are available in the European and snowball groups of cauliflower, which form curd in low temperature conditions (10°C–16°C). Thus, the present study made a novel attempt at tropicalisation of the *Pr* gene through introgression in Indian tropical cauliflower varieties to benefit the people inhabiting tropical regions. Notably, the tropical cauliflower accounts for approximately 50% to 65% of global cauliflower production (Singh and Kalia, 2021); thus, the introgressed *Pr* lines have better prospects.

Coloured vegetables are reported to be rich sources of anthocyanins, such as red cabbage (90.5 mg/100 g fw; Effat et al., 2009), black carrot (168.7 mg/100 g fw; Assous et al., 2014), and purple broccoli (572.0 mg/100 g fw; Liu et al., 2021). Other vegetables such as purple sweet potato (42.37 mg/100 g fw), red onion (29.99 mg/100 g fw), eggplant (6.31 mg/100 g fw), and red chicory (39.30 mg/100 g fw) were also reported as sources of anthocyanins (Gonçalves et al., 2021). Purple cauliflower was also reported as an excellent source of anthocyanins (375 mg/100 g fw; Chiu et al., 2010). However, Scalzo et al. (2008) reported a lower range of anthocyanin (3.38 to 7.72 mg/100 g fw) in local landraces of purple cauliflower from Italy, suggesting the prominent role of genotypes. Although the cooking processes such as boiling, steaming, and microwave cooking reduce anthocyanins in purple cauliflower ‘Graffiti’ by 42.5%, 15.7%, and 6.5%, respectively, the cooked vegetable still contains 662, 971, and 1,076 mg per 100 g dry weight basis, respectively. This suggests adequate post-cooking intake of anthocyanins to manifest their biological activity in the human body.

Overall, the level of anthocyanin in the vegetables was comparable with that found in blueberries (*Vaccinium myrtillus*; Gao and Mazza, 1994), hence suggesting dietary intake to harness health benefits associated with anthocyanins. The anthocyanin recovery rate in the human body can reach up to 12.4% (Czank et al., 2013), and their lower values of intake, i.e., 7.5 mg/day, are effective in reducing the risk of type 2 diabetes by 5% (Wedick et al., 2012).

The development of anthocyanin-rich cauliflower in tropical backgrounds will provide an option to consumers for increasing anthocyanin intake in countries like India, where most common food items are devoid of it (IFCT, 2017). The prominent anthocyanin in purple cauliflower is cyanidin 3(-coumaryl-caffeyl)glucoside-5-(malonyl)glucoside. Cyanidin is the potent anthocyanin found to have a strong anti-diabetic effect in humans (Wedick et al., 2012) and also reported to have a preventive role in cardiomyocyte apoptosis in mice (Gang et al., 2024).

Such food-based interventions are important for this region because of the high prevalence of NCDs, against which anthocyanins are considered a strong dietary agent (Gonçalves et al., 2021). Globally, anthocyanins are part of regular diets, which can be seen from the data on estimated average per day consumption of anthocyanins in Europe (31 mg), Australia (24.2 mg), China (27.6 mg), and the USA (12.5 mg) (Mesiano et al., 2024).

Both PA and PK are commercial varieties of tropical cauliflower that are being grown from June to November in northern India and from December to January in central and western India. Both varieties are rich in glucosinolates, such as sinigrin and glucoerucin (Vanlalnehi et al., 2020; Singh et al., 2025). Pusa Ashwini was reported to be low in progoitrin (Singh et al., 2025). However, both are white in colour and devoid of anthocyanin content. Thus, the anthocyanin-biofortified progenies in PA and PK backgrounds have the potential to expand the health benefits of these glucosinolate + anthocyanin cauliflower through edible curds and microgreens (Renna et al., 2020).

For a long time, conventional breeding methods have been successful in cauliflower improvement, but their process is time-consuming (up to 10 years) (Acquaah, 2015) and vulnerable to environmental factors due to the thermosensitive nature of the crop (Luhana et al., 2023). Furthermore, the *Pr* gene is semi-dominant in nature, and identification of homo- and heterozygous plants remains a challenge. This incomplete or partial dominance of the purple apical and purple curd colour delays the process of identification of homozygous purple plants in the segregating population during the introgression program. Thus, supplementing conventional breeding methods with MAS offers a reliable and effective option to reduce the time for the development of improved varieties (Gupta et al., 2010). The combined use of markers for foreground selection of the target trait and background selection for recombinant parent is suggested for the best gain in marker-assisted breeding, particularly in cases when the donor is non-agronomical and the chances of linkage drag are high. Alternatively, when the donor is an agronomically superior variety in a different maturity group, as in the present case, the combined use of foreground selection and key morphological traits results in satisfactory gain in the shortest possible period. This allows, first, the selection of morphologically desirable plants in a segregating population, followed by marker-assisted selection for the target locus. MAS has successfully facilitated introgression or pyramiding of major genes/quantitative trait loci (QTLs) for different economic traits in vegetable crops (Prasanna et al., 2015; Kalia et al., 2018). It was utilised for the biofortification of crops, such as the *Or* gene in cauliflower (Kalia et al., 2018), the high lycopene gene *og^c^* in tomato (Park et al., 2009), and the high grain protein gene *Gpc-B1* in wheat (Vishwakarma et al., 2014), which highlights the prospect of MAS in biofortification. Earlier, introgression of the *Pr* locus from Sicilian Purple into Indian cauliflower was attempted to increase anthocyanin in the curd portion (Singh et al., 2020). However, considering the taste factor and curd features of the progenies of the Sicilian Purple/cauliflower combination for the anthocyanin biofortification, it was replaced with ‘PPCF-1’, which is a true cauliflower developed using the *Pr* gene from ‘Graffiti’. PPCF-1 was used as the donor of the *Pr* gene for tropical cauliflower varieties. The present study incorporated the *Pr* gene into the genetic background of two commercial varieties of tropical cauliflower, namely, ‘PA’ and ‘PK’, using MAS combined with stringent phenotypic selection. The phenotypic selection criteria used for morphological selection were purple apical colour at the seedling stage, semi-erect leaf habit, curd maturity in October–November, purple curd, and tropical flowering habit. These traits are major differentiating traits in Indian tropical and snowball cauliflower (Singh et al., 2020). The morphological selection was effective in reducing the number of plants for screening in MAS to just 40 in F_2_ and 30 in BC_1_F_2_. The morphological selection followed by MAS was found to be effective in the present study since no linkage drag or negative impact of the *Pr* gene was noticed in either homozygous or heterozygous progenies. Similarly, no such negative impact was reported by Chiu et al. (2010) and Singh et al. (2020).

The improved progenies are superior to PA and PK for anthocyanin content when matched for days taken for developmental transitions. The progenies showed maturity in October–November, which was similar to PA and PK. However, donor ‘PPCF-1’ belongs to the late group of cauliflower, which matures in January to March. The six selected F_2:3_ progenies from PK × PPCF-1 cross and five from PA × PPCF-1 were significantly superior to the donor parent ‘PPCF-1’ (40.0 mg/100 g fw) for anthocyanin content. This could be due to the new genetic makeup, coinciding with the curding phase with a higher temperature (20°C–25°C) and greater sunlight (5.7 bright sunshine hours) than the cool temperature (10°C–16°C) and shorter duration of sunshine (3.7 bright sunshine hours) during the maturity of ‘PPCF-1’. Earlier studies also reported the roles of temperature and light factors on anthocyanin accumulation in crop genotypes (Lu et al., 2015). This highlights the better prospect of anthocyanin-biofortified cauliflower to improve public health in the tropical region.

### Genetics of purple apical and curd colour

Apical colour pigmentation in F_1_ was light purple, and in the F_2_, F_2:3_, and BC_1_F_1_ progenies, it ranged from light purple to dark purple, suggesting the role of allelic nature of the *Pr* locus (Yuan et al., 2009; Chiu et al., 2010) and/or environmental factors (Volden et al., 2009; Chen et al., 2022). In the present study, an attempt was made to segregate the individuals of segregating populations into three classes—purple, purple-green, and green apical—as suggested for the semi-dominant *Pr* gene (Chiu et al., 2010); however, the visual observations were inadequate to distinguish the plants. The observed ratio of plants did not match the ratio of 1:2:1. The single factor for purple apical and purple curd colour in Indian cauliflower agreed with the earlier report of Chiu et al. (2010). Notably, all the plants having purple apical also produced purple curd, suggesting that the same *Pr* gene regulated the purple pigmentation of both plant parts. Furthermore, the negligible difference in populations from both sets suggests the absence of the background influence of the *Pr* gene. This suggests that the *Pr* gene-regulated seedling purple apical colour can be used as a morphological marker between purple and white types as a quick progeny selection test for curd colour in cauliflower. However, the intensity of apical colour was inadequate to distinguish between homo- and heterozygous purple plants.

The purple pigmentation of apical leaves was gradually replaced by green colour, suggesting the significance of chlorophyll and anthocyanin in photoprotective and photosynthetic processes (Steyn et al., 2002). This developmental colour change pattern is contrary to the leaf pigmentation in red cabbage, wherein the leaves remain purple till the end (Yuan et al., 2009). However, the curd portion was devoid of chlorophyll; hence, it remained purple until the flowering stage, when anthocyanin degraded naturally. Thus, the *Pr* gene exhibits tissue- and stage-specific patterns of anthocyanin accumulation vis-à-vis degradation. The observations in cauliflower were contrary to those in tomato (Butelli et al., 2008). They reported the green-to-purple conversion of tomato fruits during the mature green stage.

### Morphological characteristics and developmental transitions of selected progenies

The study observed a significant difference in morphological and yield traits in 13 homozygous MAS-derived F_2:3_ progenies. The genetic recombination followed by manual selection of promising F_2_ plants performed prior to MAS could be the reason for attaining desirable yield levels in the progenies, probably due to the additive and dominant gene actions for plant weight and curd weight (Singh et al., 2020). The study highlights the significance of morphological trait-based selection for desirable-type plants in segregating populations in the rapid recovery of promising progenies in gene introgression. The promising F_2:3_ progenies for curd yield in both sets highlight their breeding use.

The intermediate position of F_1_ between Indian and snowball types indicates the quantitative nature of developmental transitions in cauliflower. The F_1_s were closer to the mid-late group of cauliflower, supporting the earlier hypothesis that the mid-late group evolved from intercross progenies between the early group of Indian cauliflower and the snowball type (Rakshita et al., 2021; Singh et al., 2020). Dey et al. (2018) also highlighted frequent introgression of genomic regions to evolve mid groups of Indian types. The observed earliness in curding and flowering in F_1_ and F_2_ than ‘PPCF-1’ but delayed than that in PA and PK suggests the quantitative nature of earliness with a dominant effect. Hulbert and Orton (1984) reported a similar trend in early flowering broccoli genotypes and Singh et al. (2020) in cauliflower/Sicilian Purple combination. The high yield of selected plants suggests that the criteria used for morphological selection were extremely effective in identifying lines with high yield potential. However, it would be interesting to see how this corresponds to retrospective genome-wide genotyping.

### Marker-assisted selection for *Pr* gene

Marker-assisted selection for a semi-dominant gene requires codominant marker(s) to distinguish visibly similar-appearing cauliflower plants. Both homozygous and heterozygous *Pr* gene-carrying plants have purple colour of seedling apical and marketable curds. The purple colour is governed by the *Pr* gene (Chiu et al., 2010), but the intensity was affected by temperature and moisture stresses (Piccaglia et al., 2002; Volden et al., 2009; Chen et al., 2022), suggesting the significance of molecular markers in the selection of homozygous plants. The present study confirmed the value of foreground selection because i) the donor parent is an agronomically superior and commercial variety in the snowball group, ii) there is no report showing linkage drag during inter-group gene transfer in cauliflower (Singh et al., 2020; Saha et al., 2021), and iii) it helped in attaining the cost-effective precision in breeding programme.

Three PCR-based molecular markers, namely, BoMYB2m, BoMYB3m, and BoMYB4m, linked to the *Pr* gene were available for breeding use (Chiu et al., 2010). The present study used these three markers, including BoMYB2m and BoMYB4m as codominant markers and one dominant marker, BoMYB3m. Both BoMYB2m and BoMYB4m could effectively distinguish 19 homozygous plants and 16 heterozygous plants. Five plants did not amplify in the first attempt by BoMYB2m; however, their repeat process for PCR analysis resulted in four as homozygous and one as a heterozygous amplicon, matching the amplification pattern of BoMYB4m. Before performing PCR analysis, the selection of plants on the basis of morphological criteria was found to be useful in selecting 40 plants in F_2_ and 30 plants in BC_1_F_2_. Furthermore, the markers were equally effective in the selection of 13 homozygous *PrPr* plants in large F_2_ populations. Singh et al. (2020) also used *BoMYB* markers while introgressing the *Pr* gene in cauliflower from Sicilian Purple. Furthermore, the MAS-selected F_2_ plants were used as recipient parent for the rapid development of cytoplasmic male sterile lines (CMS) through marker-assisted backcrossing (MABC) in the PK (of DC-23000) background (data not presented).

### Anthocyanin content in F_2_ and F_2:3_ populations

Anthocyanin accumulation was negligible in white curd cauliflower varieties ‘PA’ and ‘PK’, suggesting a biologically dormant state of the anthocyanin biosynthesis pathway. We analysed total anthocyanin content using a widely followed pH differential method, which revealed a wide variation in F_2_ populations ranging from 6.5 to 96.4 mg/100 g fw in PK × PPCF-1 and 2.34 to 97.5 mg/100 g fw in PA × PPCF-1. The observed variation in F_2_ agreed with the earlier findings of Singh et al. (2020) in the F_2_ population (0.051 to 48.42 mg/100 g fw) from white cauliflower ‘DC 466’/Sicilian Purple ‘PC-1’. Almost a similar range in purple plants from both populations could be due to the relative closeness of the recipient background since both are from the early maturity group and genetically related in the evolutionary process (Rakshita et al., 2021).

The curds of F_1_ plants showed intermediate levels of anthocyanin content, which also agreed with the earlier reported values (38.12 mg/100 g fw) (IARI, 2018; Singh et al., 2020). The state of homo- or heterozygosity of the *Pr* gene and modifiers in the recipient backgrounds may have affected the level of anthocyanin. Scalzo et al. (2008) reported a variation in anthocyanin content from 1.81 to 7.72 mg/100 g fw violet cauliflower landraces from Italy. Negligible amount of anthocyanin (<1.0 mg/100 g fw) in white curding plants agreed with the earlier findings on Pusa Snowball Kt-25 (0.19 mg/100 g fw) (IARI 2018). In white varieties, the traces of anthocyanin could be due to purple pigmentation on the curd surface due to temperature factors and anthocyanin metabolism-related structural genes (Chen et al., 2022). Higher anthocyanin content in 11 progenies could be attributed to the confirmed homozygous state of the *Pr* locus. Environmental factors also vary for the two sets because the timing of curd maturity is different in the newly derived progenies (October–November) and KTPCF-1 (end of January to February) (Luhana et al., 2023). Volden et al. (2009) and Chen et al. (2022) also reported the influence of environmental factors on anthocyanin content.

Furthermore, none of the plants in F_2_ populations and F_2:3_ progenies had anthocyanin levels higher than the earlier report value of 375.0 mg/100 g fw in ‘Graffiti’ (Chiu et al., 2010) and 182 mg/100 g fw in red cabbage (Yuan et al., 2009). This may be due to differences in genetic background and edaphic and climatic factors. However, Volden et al. (2009) reported an anthocyanin content of 73.9 ± 2.8/100 g fw in ‘Graffiti’ from Norway. The differences could be due to differences in growing conditions and tissues taken for analysis (Scalzo et al., 2008). The present study found two progenies, namely, PC2304–93 and PC2304-65, from PK × PPCF-1 and PC6704–36 and DC6704–16 from PA × PPCF-1, which are superior to the varieties reported by Volden et al. (2009). The value of anthocyanin content was higher than that of PPCF-1, which could be due to the presence of heterozygous plants (Kumar, pers. comm.). A variation in anthocyanin content in F_2_ plants was attributed to the homozygous and heterozygous states of the *Pr* locus (Butelli et al., 2008; Singh et al., 2020). Differences in the genetic makeup of the recombinants for endogenous promoters (Grotewold et al., 2000; Gonzalez et al., 2008; Chiu et al., 2010) may have affected the *Pr* allele. The occurrence of bicolour curds in segregating populations was manifested by reaction–diffusion (Turing, 1952) or the role of R2R3MYB activator gene (Ding et al., 2020; Zheng et al., 2021). This also indicates possibilities of gene conversion or epigenetic silencing in somatic cells during the mitotic division of meristems early in curd conversion in bicolour curd phenotypes. Thus, a study is underway for further investigation since the bicolour curd phenotype is a non-conventional trait in cauliflower, which may appeal to consumers.

## Conclusion

The *Pr* gene is a novel mutant in cauliflower, which imparts high anthocyanin accumulation and purple colour of the edible curd portion. The present study is the first attempt to introgress the *Pr* gene in Indian tropical cauliflower using marker-assisted selection in F_2_ and BC_1_F_1_ generations. An appreciable level of anthocyanin in heterozygous plants suggests prospects of the development of hybrids. Furthermore, the *Pr* gene did not cause any observable deformity/penalty on morphological or developmental transitions, furthering the case for commercial exploitation. The successful introgression of the *Pr* gene in the tropical cauliflower will diversify the vegetable basket with colourful, antioxidant-rich purple cauliflower.

## Data Availability

The datasets presented in this study can be found in online repositories. The names of the repository/repositories and accession number(s) can be found in the article/supplementary material.
