# Supplementary material for: Rapid recovery of homozygous Pr gene introgression lines in Indian tropical cauliflower backgrounds through combined use of morphological and molecular markers
Source: Front Plant Sci. 2025 Sep 29;16:1609917. doi: 10.3389/fpls.2025.1609917 (PMC12515921; doi:10.3389/fpls.2025.1609917)
Supplement: Supplementary file 1 [file Table1.pdf]

Rapid recovery of homozygous *Pr* gene introgression lines in Indian tropical cauliflower backgrounds through combined use of morphological and molecular markers

Shrawan Singh*[&1], Sandeep Kumar[2] and Vinay Verma[1]
[1]Division of Vegetable Science, ICAR-Indian Agricultural Research Institute, New Delhi-110012, India
[2]ICAR-Indian Agricultural Research Institute Regional Station, Katrain, Kullu, Himachal Pradesh, India-175129, India
*Corresponding author: Dr. Shrawan Singh, Principal Scientist, Division of Vegetable Science, ICAR-New Delhi-110012, India

Table S1. The *Pr* gene specific primers used for marker-assisted selection in the study.

Primer name	Sequence (5' to 3')	Amplicon size (bp)	Reference
BoMYB2$_m$ F	GTCGGAAGTGTTTAAAGAGAGAACATGAT	850/400	Chiu et al. (2010)
BoMYB2$_m$ F	TTAGAGCTATCGGGGCGTGTCTAGAGA		
BoMYB2$_m$ R	GCTTGAGGAAAAGGGTTTATATTGTGGCT		
BoMYB3$_m$ F	GGTGCATGAAGCCGTTCTCTCTTTTAATC	650/-	
BoMYB3$_m$ R	TGAAACTGAAGGAGTACTCCCTTGGTAAC		
BoMYB4$_m$ F	CCCAAGTGTATCAGCTACAGAAGAAGCCT	600/450	
BoMYB4$_m$ F	TCGTTGTTGGTTTGTCGTTCAGTGAAAT		
BoMYB4$_m$ R	AAGGAAGGTTTCTCTTTTTCATTTGGGTCTTAC		

Note: PCR reaction components and conditions:

A. PCR reaction components:

PCR reaction volume : 10 µl
Genomic DNA : 1 µl (25 ng)
PCR master mix : 5 µl (OnePCR™, GeneDirex),
Primers : 0.5 µl of forward and reverse each (25µM)
Sterile double-distilled water: 3 µl

B. PCR conditions:

- Hot start: 104 ºC;
- PCR cycle:
- Step 1: one cycle of initial denaturation: 94 ºC for 2 min;
- Step II: 35 cycles: a. Denaturation: 94 ºC for 45 sec; b. Annealing: 54 ºC for 45 sec; c. Extension at 72 ºC for 45 sec;
- Step III: one cycle for final elongation at 72 ºC for 7 min
- Hold : at 4 ºC for 30 min.
