# Supplementary material for: Rapid recovery of homozygous Pr gene introgression lines in Indian tropical cauliflower backgrounds through combined use of morphological and molecular markers
Source: Front Plant Sci. 2025 Sep 29;16:1609917. doi: 10.3389/fpls.2025.1609917 (PMC12515921; doi:10.3389/fpls.2025.1609917)
Supplement: Supplementary file 2 [file Table2.pdf]

Rapid recovery of homozygous *Pr* gene introgression lines in Indian tropical cauliflower backgrounds through combined use of morphological and molecular markers

Shrawan Singh*[&1], Sandeep Kumar[2] and Vinay Verma[1]

[1]Division of Vegetable Science, ICAR-Indian Agricultural Research Institute, New Delhi-110012, India

[2]ICAR-Indian Agricultural Research Institute Regional Station, Katrain, Kullu, Himachal Pradesh, India-175129, India

*Corresponding author: Dr. Shrawan Singh, Principal Scientist, Division of Vegetable Science, ICAR-New Delhi-110012, India

Table S2. Morphological characters, yield traits and days to developmental transitions of MAS-derived homozygous $F_{2:3}$ progenies for the *Pr* gene in Indian tropical cauliflower.

Genotypes	Morphological traits				Yield traits			Days to developmental transitions						
	Leaf length (cm)	Leaf width (cm)	Plant height (cm)	Plant spread (cm)	Gross plant weight (g)	Marketable curd weight (g)	Marketable curd weight (g)	Seedl stage	Curd initiation	Curd maturity	Bolting	Flowering	Flowering termination	Seed harvesting
Parents/F_1s														
Pusa Kartiki	48.9±4.0^{a}	14.9±1.3^{ef}	64.0±3.5^{a}	69.5±6.6^{a}	1534.3±80.2^{b}	566.7±60.3^{abcde}	17.8±1.9^{abcde}	34^{b}	100.7±3.2^{k}	113.8±2.1^{j}	131.4±3.2^{klm}	147.7±3.2^{m}	187.3±2.1^{l}	231.0±3.[illegible]^{hi}
Pusa Ashwini	44.5±2.4^{abcd}	16.2±1.0^{de}	56.9±3.9^{bc}	68.2±2.6^{a}	1267.0±41.7^{cd}	580.0±36.1^{abcd}	18.3±1.1^{abcd}	34^{b}	106.7±2.5^{i}	118.7±2.5^{i}	139.3±2.5^{hi}	159.7±2.5^{hij}	192.0±1.7^{k}	242.3±2.5^{e}
PPCF-1	41.0±6.2^{cde}	20.7±1.3^{ab}	51.2±4.7^{cdefgh}	50.7±2.7^{bc}	1025.3±45.5^{ghi}	528.0±37.0^{cdefg}	16.6±1.2^{cdefg}	65^{a}	188.7±2.5^{b}	207.2±4.2^{b}	226.3±2.5^{b}	243.6±2.5^{c}	283.3±4.2^{b}	NA
PK/PPCF-1	43.8±2.5^{abcd}	12.4±1.1^{gh}	51.0±4.1^{cdefgh}	54.8±2.8^{b}	1340.7±48.8^{c}	623.0±35.7^{ab}	19.6±1.1^{ab}	34^{b}	136.3±3.5^{e}	148.3±3.1^{e}	165.4±3.2^{e}	177.7±2,3^{e}	202.0±5.3^{hi}	243.0±2.6^{e}
PA/PPCF-1	44.5±3.9^{abcd}	15.4±0.9^{e}	53.3±5.0^{cdefg}	45.9±2.3^{cd}	1177.7±118.2^{def}	484.7±67.2^{efgh}	15.3±2.1^{efgh}	34^{b}	98.7±2.3^{l}	110.0±3.0^{kl}	133.3±2.9^{jkl}	155.3±2.9^{k}	215.7±3.2^{e}	232.0±4.0^{gh}
$F_{2:3}$ progenies (PA/PPCF-1)														
PC6704-35	40.2±2.8^{de}	17.3±0.8^{cd}	54.2±3.5^{cdef}	41.1±3.7^{de}	1162.7±47.9^{def}	503.3±62.3^{defgh}	15.8±2.0^{defgh}	34^{b}	97.7±3.1^{lm}	112.3±3.1^{jk}	129.3±2.9^{mn}	142.0±4.0^{n}	180.1±3.1^{m}	224.7±4.2^{jk}
PC6704-36	37.8±2.4^{e}	13.5±0.9^{fg}	43.3±2.9^{i}	38.0±2.7^{ef}	1267.7±56.1^{cd}	586.0±51.4^{abcd}	18.4±1.6^{abcd}	34^{b}	101.0±2.6^{k}	118.0±3.6^{i}	136.7±2.5^{ij}	160.3±2.9^{hi}	195.7±3.2^{j}	235.3±3.1^{fg}
PC6704-2	42.1±3.2^{bcde}	14.6±0.6^{ef}	51.5±3.4^{cdefgh}	34.7±2.4^{f}	1150.0±123.5^{defg}	538.0±13.1^{bcdefg}	16.9±0.4^{bcdefg}	34^{b}	104.0±2.6^{j}	118.2±3.1^{i}	136.1±2.9^{ij}	156.2±3.2^{jk}	200.3±4.2^{i}	228.7±4.0^{hij}
PC6704-11	36.8±1.6^{e}	16.1±1.0^{de}	47.6±5.9^{fghi}	41.6±1.2^{de}	1097.3±45.8^{efgh}	545.0±27.8^{bcdef}	17.2±0.9^{bcdef}	34^{b}	118.0±3.0^{f}	133.7±2.5^{f}	159.0±3.6^{f}	167.3±4.0^{g}	207.7±2.1^{g}	253.0±3.6^{d}
PC6704-34	48.4±3.1^{a}	15.8±0.6^{de}	55.0±3.7^{cd}	43.9±4.2^{de}	941.3±72.9^{i}	439.3±85.1^{h}	13.8±2.7^{h}	34^{b}	102.0±2.6^{k}	114.3±3.1^{j}	134.0±1.7^{jk}	158.0±4.0^{ijk}	208.0±4.0^{g}	224.7±4.0^{jk}
PC6704-16	42.1±2.0^{bcde}	18.9±1.2^{bc}	55.3±1.7^{bcd}	52.3±2.7^{b}	1106.3±52.8^{efgh}	598.0±58.9^{abc}	18.8±1.9^{abc}	34^{b}	96.0±2.6^{m}	109.3±2.5^{l}	127.7±5.0^{n}	145.6±2.3^{m}	207.3±2.3^{g}	226.0±3.6^{ijk}
$F_{2:3}$ progenies (PK/PPCF-1)														
PC2304-66	37.9±3.4^{e}	12.3±0.8^{gh}	48.0±3.0^{efghi}	45.2±4.2^{cd}	1140.3±94.5^{defg}	502.7±30.7^{defgh}	15.8±1.0^{defgh}	34^{b}	98.7±1.5^{l}	113.3±2.5^{j}	130.0±3.0^{lmn}	151.0±3.0^{l}	193.0±2.0^{jk}	230.3±5.5^{b}
PC2304-21	46.3±2.0^{abc}	11.2±0.6^{hi}	56.1±5.3^{bc}	53.7±6.8^{b}	1574.7±59.4^{ab}	651.0±79.8^{a}	20.5±2.5^{a}	34^{b}	105.7±3.2^{ij}	117.0±2.0^{i}	139.7±3.2^{hi}	164.7±3.2^{g}	211.7±1.5^{f}	229.7±4.0^{hij}
PC2304-65	46.9±1.5^{ab}	12.7±1.4^{gh}	45.7±2.7^{hi}	50.9±4.2^{bc}	1002.3±45.5^{hi}	459.7±44.1^{fgh}	14.5±1.4^{fgh}	34^{b}	106.7±1.5^{i}	123.0±2.6^{h}	143.0±5.5^{h}	161.3±5.5^{h}	194.7±0.6^{jk}	242.0±2.6^{e}
PC2304-64	41.1±4.2^{cde}	12.0±0.5^{ghi}	48.9±5.9^{defghi}	43.7±3.6^{de}	1053.7±84.3^{fghi}	600.3±12.7^{abc}	18.9±0.4^{abc}	34^{b}	118.0±2.6^{f}	135.0±2.6^{f}	155.0±2.3^{g}	172.7±2.3^{f}	200.7±2.3^{i}	230.7±4.0^{ghi}
PC2304-35	37.0±3.2^{e}	10.2±0.8^{i}	43.7±3.5^{i}	44.7±4.6^{cd}	959.7±83.8^{i}	453.0±89.1^{gh}	14.3±2.8^{gh}	34^{b}	114.0±2.6^{g}	128.7±1.5^{g}	143.0±2.6^{h}	157.0±2.6^{jk}	203.0±2.6^{hi}	223.3±2.5^{k}
PC2304-61	40.0±3.3^{de}	12.3±1.8^{gh}	47.3±3.9^{ghi}	37.8±3.4^{ef}	1146.7±118.5^{defg}	505.7±26.5^{defgh}	15.9±0.8^{defgh}	34^{b}	110.3±3.5^{h}	125.3±3.8^{h}	154.3±2.3^{g}	175.7±2.3^{ef}	204.7±3.1^{h}	238.7±2.3^{ef}
PC2304-93	41.5±1.7^{bcde}	13.0±0.5^{fgh}	54.6±1.4^{cde}	42.6±2.3^{de}	1213.0±37.2^{cde}	546.7±26.6^{bcde}	17.2±0.8^{bcde}	34^{b}	148.7±2.5^{d}	162.3±4.2^{d}	173.7±2.5^{d}	193.7±2.5^{d}	240.0±2.0^{d}	263.0±2.6^{c}
Graffiti	43.8±4.0^{abcd}	18.8±1.2^{bc}	48.8±3.3^{defghi}	53.5±2.9^{b}	1538.3±98.7^{b}	620.0±56.0^{ab}	19.5±1.8^{ab}	65^{a}	199.4 2.3^{a}	225.0±2.6^{a}	243.7±3.5^{a}	266.3±3.1^{a}	289.5±1.5^{a}	NA

Significant pairwise differences between lines ($p<0.05$) are denoted by values followed by different letters.
