# Supplementary material for: Rapid recovery of homozygous Pr gene introgression lines in Indian tropical cauliflower backgrounds through combined use of morphological and molecular markers
Source: Front Plant Sci. 2025 Sep 29;16:1609917. doi: 10.3389/fpls.2025.1609917 (PMC12515921; doi:10.3389/fpls.2025.1609917)
Supplement: Supplementary file 3 [file Image1.pdf]

Rapid recovery of homozygous *Pr* gene introgression lines in Indian tropical cauliflower backgrounds through combined use of morphological and molecular markers

Shrawan Singh*[&1], Sandeep Kumar[2] and Vinay Verma[1]

[1]Division of Vegetable Science, ICAR-Indian Agricultural Research Institute, New Delhi-110012, India

[2]ICAR-Indian Agricultural Research Institute Regional Station, Katrain, Kullu, Himachal Pradesh, India-175129, India

*Corresponding author: Dr. Shrawan Singh, Principal Scientist, Division of Vegetable Science, ICAR-New Delhi-110012, India

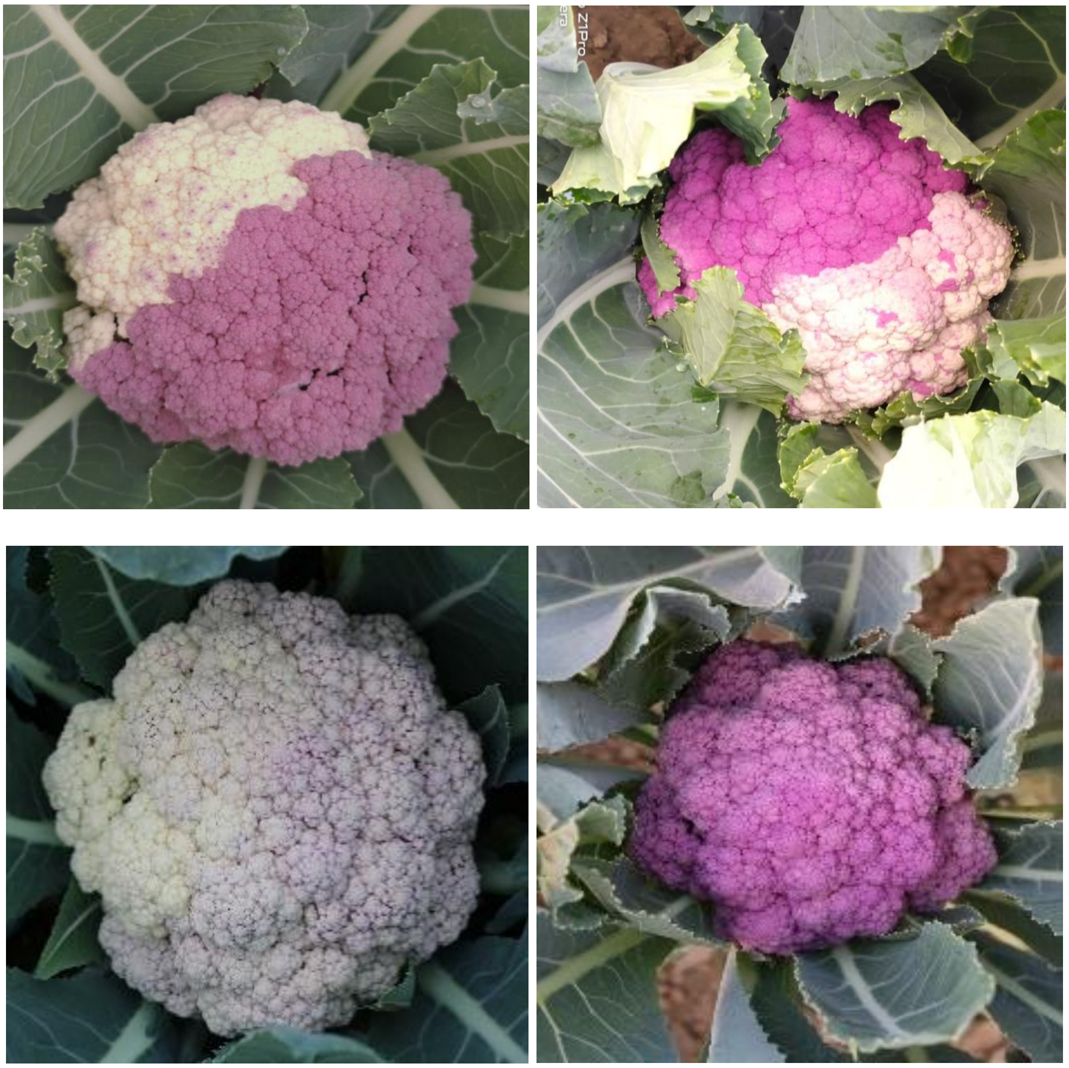

Fig. S1. Bicolour pattern observed in segregating populations of PA/PPCF-1 and PK/PPCF-1.
