# Supplementary material for: Rapid recovery of homozygous Pr gene introgression lines in Indian tropical cauliflower backgrounds through combined use of morphological and molecular markers
Source: Front Plant Sci. 2025 Sep 29;16:1609917. doi: 10.3389/fpls.2025.1609917 (PMC12515921; doi:10.3389/fpls.2025.1609917)
Supplement: Supplementary file 4 [file Image2.pdf]

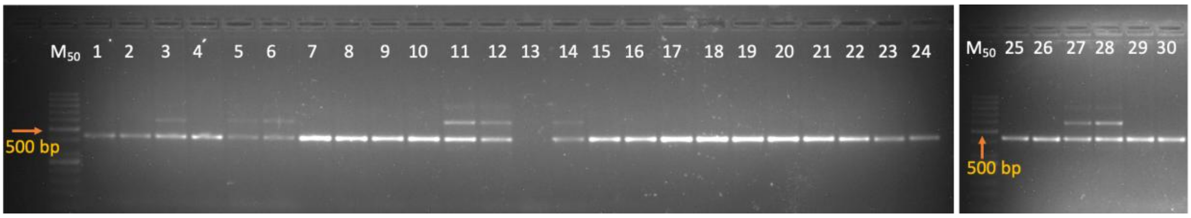

Fig. S2. Screening of *Pr* gene-specific primers BoMYB4m (Chiu et al. 2010) in BC_1F_2 populations from PA × PPCF-1 (Sl. No. 1-18 plants), PK × PPCF-1 (Sl. No. 19-30 plants). M_{50} is a marker of 50 base pair.
